# A Compound Feed Additive Improves Saline–Alkaline Stress Tolerance in Nile Tilapia (*Oreochromis niloticus*) Through Regulation of Hepatic Metabolism, Osmoregulation, and Intestinal Health

**DOI:** 10.3390/ani16132073

**Published:** 2026-07-05

**Authors:** Jinquan Fan, Yuxi Yan, Yuxing Huang, Liqiao Chen, Xiaodan Wang

**Affiliations:** Laboratory of Aquaculture Nutrition and Environmental Health, School of Life Sciences, East China Normal University, Shanghai 200241, China; 20163602059@m.scnu.edu.cn (J.F.); m18774834867@163.com (Y.Y.); hfysr@163.com (Y.H.); lqchen@bio.ecnu.edu.cn (L.C.)

**Keywords:** Nile tilapia, saline–alkaline stress, compound feed additive, energy metabolism, osmoregulation

## Abstract

Saline–alkaline aquaculture is a promising strategy to alleviate freshwater shortages; however, such environments severely impair fish growth and physiological homeostasis. Nutritional regulation has been proposed to improve stress tolerance, yet the benefits of single additives are often limited under the multifactorial challenges imposed by saline–alkaline conditions. Therefore, a compound feed additive (CFA) consisting of glutamate, cholesterol, β-glucan, myo-inositol, zinc methionine, and curcumin was developed and evaluated in Nile tilapia (*Oreochromis niloticus*). To assess the robustness and practical applicability of this nutritional strategy, three independent feeding trials were conducted using different commercial basal diets as validation systems. Within each dietary system, fish were reared under freshwater (FW), saline–alkaline water (SAW), or saline–alkaline water supplemented with CFA (SAW+CFA). Saline–alkaline stress significantly reduced WG and SR, increased FCR, and elevated VSI and HSI, indicating impaired growth performance and metabolic burden. These changes were accompanied by increased serum glucose and ion concentrations (Na^+^, K^+^, Cl^−^), elevated ammonia levels, and reduced crude protein content. Dietary CFA improved growth and feed utilization under saline–alkaline conditions. It enhanced hepatic glycogen content and reduced serum glucose levels. Meanwhile, it downregulated glycolysis-related genes (*hk*, *pfk1*, *pk*) and upregulated genes involved in gluconeogenesis and the pentose phosphate pathway (*g6pase*, *pc*, *g6pdh*), indicating altered glucose metabolism and improved energy homeostasis. Saline–alkaline stress induced oxidative stress, apoptosis, and histological damage in the liver, whereas CFA alleviated these alterations by reducing MDA levels, enhancing antioxidant enzyme activities (CAT, GSH-Px, T-SOD) and suppressing apoptosis-related genes (*caspases*, *p53*, *c-myc*). In addition, CFA alleviated saline–alkaline stress-induced gill structural damage and reduced serum ion concentrations while modulating ion transport-related gene expression, suggesting improved osmoregulatory capacity. It also enhanced ammonia metabolism and transport, as reflected by reduced serum ammonia levels and altered expression of related genes. Furthermore, Saline–alkaline stress impaired intestinal structure and function, whereas CFA improved intestinal villus structure, increased digestive enzyme activities (amylase, trypsin, lipase), and suppressed pro-inflammatory genes (*il-1β*, *il-8*). Importantly, similar beneficial response patterns were observed across the three independently analyzed dietary systems. Overall, CFA improved saline–alkaline adaptability of Nile tilapia and was associated with improvements in energy metabolism, oxidative homeostasis, osmoregulation, ammonia detoxification, and intestinal function, providing a practical nutritional strategy for saline–alkaline aquaculture.

## Simple Summary

Saline–alkaline aquaculture offers an important approach to expanding fish production in regions facing freshwater shortages. However, saline–alkaline environments can negatively affect fish growth and health. In this study, we developed a compound feed additive (CFA) containing six functional ingredients with complementary physiological roles and evaluated its effects in Nile tilapia cultured under saline–alkaline conditions. To assess the robustness of this nutritional strategy, the additive was tested in three independent feeding trials using different commercial basal diets. The results showed that CFA improved growth performance and feed utilization while enhancing antioxidant capacity, osmoregulation, ammonia metabolism, and intestinal health. Similar beneficial responses were observed across different dietary backgrounds, suggesting that the effectiveness of CFA is not restricted to a specific feed formulation. These findings suggest that a functionally integrated nutritional strategy may improve the adaptability of Nile tilapia to saline–alkaline environments and may provide practical support for the sustainable development of saline–alkaline aquaculture.

## 1. Introduction

Aquaculture is a major contributor to global food security, providing high-quality animal protein and essential micronutrients to a growing human population [1,2]. However, expansion of the industry in response to rising consumer demand has intensified pressure on freshwater resources. This trend, compounded by climate change, infrastructure development, and industrial pollution, has led to increasing freshwater scarcity and progressive salinization and alkalization of available water sources [3,4]. These changes pose substantial constraints on conventional freshwater aquaculture systems. Under these circumstances, the development and utilization of saline–alkaline water resources have emerged as a strategic priority for sustainable aquaculture [5,6]. Saline–alkaline environments are physiochemically complex and can profoundly disrupt the survival and physiological homeostasis of aquatic animals [7,8]. Exposure often results in suppressed growth, impaired antioxidant and immune function, metabolic disturbances, and dysregulation of ion transport [9]. In severe cases, compromised ammonia excretion may lead to ammonia intoxication and mortality. Saline–alkaline water is typically characterized by elevated salinity and high carbonate alkalinity [10]. The associated high pH and complex ionic composition can interact through multiple physiological pathways, amplifying environmental stress [11,12]. Thus, mitigating the toxic effects of saline–alkaline conditions represent a central challenge for the successful development of saline–alkaline aquaculture.

Nutritional intervention is widely regarded as a feasible and promising strategy to mitigate saline–alkaline stress [13,14]. Under saline–alkaline conditions, fish must expend additional energy to maintain osmotic and acid–base homeostasis, which is often accompanied by increased ion transport demand, as well as elevated oxidative stress and impaired immune function in fish [15]. However, most previous studies have focused on single-function nutritional interventions, whose regulatory effects are often limited to specific physiological processes [16,17]. For instance, antioxidant supplements such as vitamins or plant-derived compounds may alleviate oxidative stress, but their effects on ion homeostasis and metabolic regulation are often limited under saline–alkaline conditions [18]. Similarly, Osmolytes such as betaine or taurine are widely reported to enhance osmotic regulation and cellular stability under salinity stress; however, their roles in regulating energy metabolism and ammonia detoxification appear to be limited or indirect [19]. In addition, probiotics and immunostimulants have been widely reported to enhance immune responses and stress resistance in fish; however, their effects on the coordinated regulation of energy metabolism and osmoregulation under saline–alkaline conditions remain limited [20,21]. Collectively, these findings indicate that single-function additives are insufficient to simultaneously meet the integrated demands for energy supply, ion regulation, and metabolic homeostasis under saline–alkaline stress [22]. Therefore, strategies targeting a single physiological pathway remain insufficient to comprehensively improve the adaptive capacity of fish under saline–alkaline stress.

Given that saline–alkaline conditions simultaneously disrupt energy metabolism, osmotic balance, ion transport, antioxidant defense, and immune function, a functionally integrated nutritional strategy is required. In this study, a composite feed additive was designed to target these interconnected physiological processes through complementary functional components. Glutamate was included to support energy metabolism and provide metabolic substrates while contributing to redox regulation under stress conditions [23,24]. Inositol functions as an important osmolyte, helping to maintain cellular osmotic balance and membrane stability under hyperosmotic environments [25,26]. β-glucan was incorporated to enhance innate immune responses and improve disease resistance under stress [27]. Cholesterol plays a critical role in maintaining membrane integrity and modulating ion transport processes, which are essential for osmoregulation [28]. Zinc-methionine contributes to antioxidant defense and protein metabolism, thereby supporting cellular protection and metabolic stability [29]. Curcumin, a natural bioactive compound, was included for its antioxidant and anti-inflammatory properties, which help alleviate oxidative damage and inflammatory responses under stress [30]. Collectively, these components were selected to target multiple physiological disturbances induced by saline–alkaline stress, including disruptions in energy metabolism, osmotic balance, antioxidant defense, immune function, and intestinal integrity. Such a coordinated approach may overcome the limitations of single-nutrient interventions and ultimately improve the overall physiological adaptability of fish.

Nile tilapia (*Oreochromis niloticus*) is one of the most economically important aquaculture species worldwide and exhibits relatively high tolerance to salinity and alkalinity, making it a suitable model for studies of saline–alkaline adaptation [9,31]. In addition, its metabolic plasticity and carbohydrate utilization capacity provide a useful framework for evaluating nutritional intervention strategies [32]. In the present study, we systematically evaluated the effects of a compound feed additive on saline–alkaline adaptability in Nile tilapia. By assessing growth performance and key physiological health indicators, with particular emphasis on hepatic metabolism, intestinal integrity, and gill-mediated osmoregulation, this study aimed to investigate the regulatory effects of the compound additive on saline–alkaline adaptation in Nile tilapia and to provide a scientific basis for the development of functional feeds for saline–alkaline aquaculture systems. Collectively, these components were selected to target multiple physiological disturbances induced by saline–alkaline stress, including disruptions in energy metabolism, osmotic balance, antioxidant defense, immune function, and intestinal integrity. This design was based on the hypothesis that integrating functionally complementary components could provide a broader and more effective adaptive response than single-function nutritional interventions, thereby improving the overall physiological adaptability of fish.

## 2. Materials and Methods

### 2.1. Diet Preparation

The CFA consisted of glutamate (7.79 g/kg), cholesterol (8.30 g/kg), β-glucan (0.50 g/kg), inositol (0.401 g/kg), zinc methionine (0.30 g/kg), and curcumin (0.20 g/kg) (Table 1). The inclusion levels of each component were selected based on effective dosage ranges reported in previous studies on teleost fish. The CFA was incorporated into three commercially available basal diets (TW, HX, and DBN) according to the experimental design to produce the test diets. Although the three basal feeds were obtained from different manufacturers, namely TongWei (TW), HengXing (HX), and DaBeiNong (DBN), their crude protein and lipid contents were broadly comparable (Table 2). These commercial diets were selected because they represent different feed formulations commonly used in Nile tilapia farming. The inclusion of multiple commercial basal diets allowed us to evaluate whether the effects of CFA were consistent across different feed formulations and manufacturing backgrounds, thereby improving the practical relevance and general applicability of the findings. Accordingly, three independent feeding trials were conducted using the three commercial basal diets as separate validation systems. The objective was not to compare feed brands, but to examine whether similar response patterns to CFA could be observed across different dietary backgrounds. Therefore, each dietary system was treated as an independent experiment and analyzed separately. The commercial basal diets were first ground into fine powder before the CFA was added. The CFA was then thoroughly mixed with the powdered feed to ensure homogeneity prior to pelleting. Importantly, all FW, SAW, and SAW+CFA diets underwent identical processing procedures, including grinding, mixing, and extrusion under the same conditions to ensure that no processing-related differences existed among treatments. All experimental diets were processed into sinking pellets using an F-26 II twin-screw extruder. The pellets were air-dried to a moisture content below 10% and stored at −20 °C until use.

### 2.2. Experimental Animals and Rearing Conditions

Juvenile Nile tilapia (*Oreochromis niloticus*) was obtained from a commercial aquaculture farm. The initial body weight was 3.46 ± 0.04 g. Fish were acclimated for two weeks at 28 ± 1 °C prior to the experiment. A total of 675 healthy fish were randomly distributed into 27 tanks (9 treatment groups × 3 replicates), with 25 fish per tank. Saline–alkaline challenge was established gradually. Salinity was increased daily by 3–4 ppt and alkalinity by 0.5 g/L until reaching 16 ppt salinity and 2.5 g/L alkalinity. No abnormal mortality was observed during the transition period. The formal feeding trial lasted 42 days. Water temperature was maintained at 28 ± 1 °C, dissolved oxygen remained above 7.0 mg L^−1^, and a 12 h light/12 h dark photoperiod was applied. Continuous aeration was provided throughout the experiment. Water quality parameters and feed intake were recorded daily.

### 2.3. Sample Collection

At the end of the trial, fish were fasted for 24 h before sampling. Individual body weight and total length were measured. Liver and viscera were dissected and weighed to calculate the hepatosomatic index (HSI) and viscerosomatic index (VSI). Growth performance parameters included weight gain (WG), specific growth rate (SGR), survival rate (SR), condition factor (CF), and feed conversion ratio (FCR). Two fish per tank were randomly selected for whole-body composition analysis and stored at −20 °C. Four fish per tank were sampled for caudal vein blood collection. Blood samples were centrifuged at 4 °C and 3500 rpm for 10 min to obtain serum for osmolality and biochemical analyses. Liver, gill, and intestinal tissues were rapidly frozen in liquid nitrogen and stored at −80 °C for subsequent analyses.

### 2.4. Proximate Composition Analysis

Proximate composition of whole fish and diets was determined according to AOAC (1995) procedures. Moisture content was measured by oven-drying, crude lipid by Soxhlet extraction, crude protein by the Kjeldahl method (Kjeltec™ 8200, Foss, Hillerød, Denmark), and ash content by combustion in a muffle furnace at 550 °C.

### 2.5. Histological Analysis

Three fish per tank were randomly selected for histological examination. Gill, liver, and intestinal tissues were fixed in 4% paraformaldehyde for 48 h, embedded in paraffin, sectioned at 5 μm thickness, and stained with hematoxylin and eosin (H&E). Histological observations were conducted using an Olympus BX51 light microscope (Olympus, Tokyo, Japan) for qualitative assessment of tissue morphology.

### 2.6. Serum and Tissue Biochemical Analyses

Serum osmolality was measured using a freezing-point osmometer (Model 210, Advanced Instruments, Norwood, MA, USA). Other serum biochemical parameters were determined using commercial diagnostic kits (Nanjing Jiancheng Bioengineering Institute, Nanjing, China) following the manufacturer’s instructions. Serum glucose (A154-1-1), Cl^−^ (C003-2-1), Na^+^ (C002-1-1), K^+^ (C001-2-1), and ammonia (A086-2-1) levels were analyzed. For tissue analyses, liver, gill, and intestinal samples were homogenized in ice-cold physiological saline at a ratio of 1:9 (*w*/*v*) and centrifuged at 4 °C and 2500 rpm for 10 min. The supernatants were collected for determination of total antioxidant capacity (T-AOC, A015-2-1), catalase (CAT, A007-1-1), total superoxide dismutase (T-SOD, A001-3-2), glutathione peroxidase (GSH-Px, A005-1-2), malondialdehyde (MDA, A003-1-2), reduced glutathione (GSH, A006-2-1), total protein (TP, A045-2-2), glutamate (Glu, A074-1-1), glutamine synthetase (GS, A047-1-1), glycogen content (A043-1-1), and digestive enzyme activities (α-amylase C016-1-2, trypsin A080-2-2, and lipase A054-1-1).

### 2.7. Quantitative Real-Time PCR Analysis

Total RNA was extracted from liver, kidney, and gill tissues using RNAiso Plus (Takara, Bio Inc., Kusatsu, Shiga, Japan). RNA quality and concentration were assessed by agarose gel electrophoresis and NanoDrop 2000 spectrophotometry (Thermo Fisher Scientific, Waltham, MA, USA). First-strand cDNA was synthesized using HiScript III RT SuperMix (Vazyme, Nanjing, China). Quantitative real-time PCR (qPCR) was performed on a CFX96 Real-Time PCR System (Bio-Rad Laboratories, Hercules, CA, USA) using ChamQ Universal SYBR qPCR Master Mix (Vazyme, Nanjing, China). β-actin and EF-1α were used as reference genes after validation of expression stability. Relative gene expression levels were calculated using the 2^−ΔΔCt^ method. Primer sequences are listed in Table 3, All RT-qPCR reactions were performed using the same thermal cycling conditions for all target genes.

### 2.8. Statistical Analysis

Data are presented as mean ± standard deviation (SD). Statistical analyses were performed using SPSS Statistics 20.0. After confirming normality and homogeneity of variance, the three feeding trials were analyzed separately because they were designed as independent validation experiments using different commercial basal diets. Feed brand was not considered an experimental factor, and the objective of the study was not to compare feed formulations but to determine whether the efficacy of CFA could be consistently observed across different dietary backgrounds. Therefore, no between-brand comparisons or feed × treatment interaction analyses were conducted. Within each dietary system, independent-samples *t*-tests were used to compare FW vs. SAW and SAW vs. SAW+CFA groups. Statistical significance was set at * *p* < 0.05, ** *p* < 0.01, and *** *p* < 0.001. The rearing tank was considered the experimental unit for all statistical analyses. When multiple fish were sampled from the same tank, measurements were average at the tank level prior to statistical analysis. Therefore, each treatment group consisted of three independent biological replicates corresponding to the three replicate tanks (*n* = 3).

## 3. Result

### 3.1. Growth Performance

**Figure 1:**
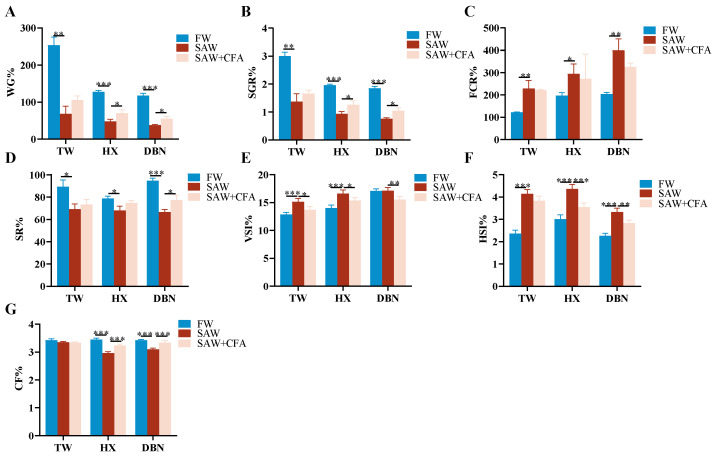
Effects of the compound feed additive on growth performance indices of Nile tilapia under saline–alkaline stress: (**A**) weight gain rate (WG); (**B**) specific growth rate (SGR); (**C**) feed conversion ratio (FCR); (**D**) survival rate (SR); (**E**) viscerosomatic index (VSI); (**F**) hepatosomatic index (HSI); (**G**) condition factor (**C**,**F**). FW represents the freshwater group, SAW represents the saline–alkaline water group, and SAW+CFA represents the saline–alkaline water group supplemented with compound feed additives. Data are presented as the mean ± SD (*n* = 3). Differences among different treatment groups under the same basal diet were analyzed by independent-sample *t*-test. Significance levels are indicated as * *p* < 0.05, ** *p* < 0.01, *** *p* < 0.001.

Saline–alkaline stress significantly affected the growth performance and somatic indices of Nile tilapia. Compared with the FW group, the SAW treatment significantly reduced weight gain (WG) and specific growth rate (SGR) under all three commercial basal diets (TW, HX, and DBN) (Figure 1A,B), accompanied by a significant increase in feed conversion ratio (FCR) (Figure 1C). In addition, SAW treatment markedly decreased survival rate (SR) (Figure 1D). Under saline–alkaline conditions, supplementation with the compound feed additive (SAW+CFA) significantly improved growth performance across all basal diet groups. Specifically, compared with the SAW group, WG increased by approximately 46–54%, survival increased by approximately 6–16%, and FCR showed a consistent decreasing trend across the three commercial basal diet systems (Figure 1A–D). For the somatic indices, SAW treatment significantly increased viscerosomatic index (VSI) and hepatosomatic index (HSI), whereas SAW+ CFA treatment significantly reduced both VSI and HSI in all three diet groups (Figure 1E,F). The condition factor (CF) exhibited relatively minor variation among treatments overall; however, SAW treatment significantly decreased CF in some diet groups (Figure 1G), and SAW+CFA supplementation partially restored this reduction.

### 3.2. Whole Body Composition Analysis

**Figure 2:**
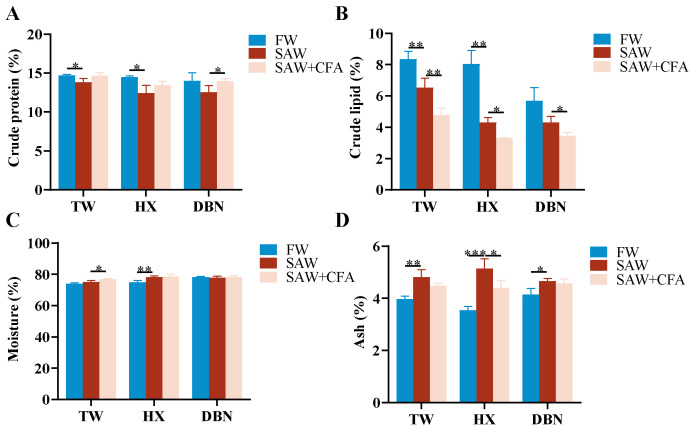
Effects of the compound feed additive on proximate composition of whole body in Nile tilapia under saline–alkaline stress: (**A**) crude protein content; (**B**) crude lipid content; (**C**) moisture content; (**D**) ash content. FW represents the freshwater group, SAW represents the saline–alkaline water group, and SAW+CFA represents the saline–alkaline water group supplemented with compound feed additives. Data are presented as the mean ± SD *(n* = 3). Significant differences were analyzed by independent-sample *t*-test. Differences among different treatment groups under the same basal diet were analyzed by independent-sample *t*-test. Significance levels are indicated as * *p* < 0.05, ** *p* < 0.01, *** *p* < 0.001.

Different water environments and dietary treatments exerted certain effects on the whole-body composition of Nile tilapia. Compared with the FW group, the SAW treatment resulted in a reduction in crude protein content at all three experimental sites (Figure 2A), whereas the SAW+CFA treatment partially alleviated this declining trend. Crude lipid content was significantly decreased under SAW treatment (Figure 2B), and the SAW+CFA group generally maintained a relatively low level. Meanwhile, both SAW and SAW+CFA treatments slightly increased whole-body moisture content (Figure 2C). In addition, SAW treatment significantly elevated whole-body ash content (*p* < 0.05; Figure 2D). Although the crude ash level in the SAW+CFA group was lower than that in the SAW group, it remained higher than that in the FW group.

### 3.3. Hepatic Energy Metabolism

**Figure 3:**
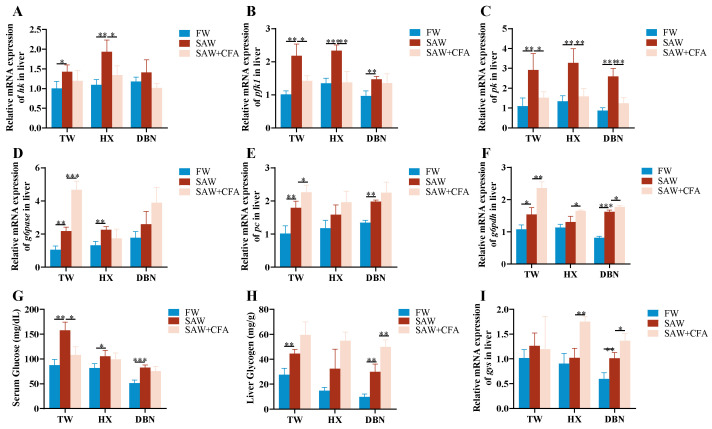
Effects of the compound feed additive on hepatic energy metabolism-related gene expression and metabolic parameters of Nile tilapia under saline–alkaline stress: (**A**–**F**) relative mRNA expression levels of glycolysis and gluconeogenesis-related genes (**A**) *hk*, (**B**) *pfk1*, (**C**) *pk*, (**D**) *g6pase*, (**E**) *pc*, and (**F**) *g6pdh* in the liver; (**G**) serum glucose content; (**H**) hepatic glycogen content; (**I**) relative mRNA expression level of hepatic *gys* gene. Gene expression was determined by qRT-PCR and normalized using β-actin and EF-1α as internal reference genes. FW, SAW, and SAW+CFA groups are defined as previously described. Data are presented as the mean ± SD (*n* = 3). Significant differences were analyzed by independent-sample *t*-test. Differences among different treatment groups under the same basal diet were analyzed by independent-sample *t*-test. Significance levels are indicated as * *p* < 0.05, ** *p* < 0.01, *** *p* < 0.001.

To evaluate the effects of different treatments on hepatic energy metabolism and glucose metabolic pathways in fish, the present study analyzed the expression levels of genes involved in glycolysis, gluconeogenesis, and glycogen synthesis in liver tissue. Serum glucose concentration and hepatic glycogen content were also determined. Regarding glycolysis-related genes, the mRNA expression levels of hepatic *hk*, *pfk1*, and *pk* in the SAW group were significantly higher than those in the FW group. In contrast, their expression levels in the SAW+CFA group were downregulated to varying degrees compared with the SAW group, with more pronounced effects observed under the TW and HX dietary conditions (Figure 3A–C).

For gluconeogenesis and related metabolic pathways, the transcriptional levels of *g6pase* and *pc* were significantly elevated in the SAW group. The SAW+CFA treatment further upregulated these genes to varying extents under different dietary conditions (Figure 3D,E). In addition, the mRNA expression of *g6pdh*, a key enzyme in the pentose phosphate pathway, was significantly higher in the SAW group than in the FW group and was further increased following SAW+CFA supplementation (Figure 3F). Consistent with these gene expression changes, serum biochemical analysis showed that serum glucose levels in the SAW group were significantly higher than those in the FW group. SAW+CFA treatment significantly reduced blood glucose levels under certain dietary conditions (Figure 3G). Meanwhile, hepatic glycogen content in the SAW+CFA group was significantly higher than that in both the FW and SAW groups, indicating enhanced glycogen synthesis capacity (Figure 3H). Furthermore, hepatic *gys* mRNA expression was markedly upregulated in the SAW+CFA group, further supporting the enhanced glycogen synthesis observed (Figure 3I).

### 3.4. Histological Observation of the Liver

**Figure 4:**
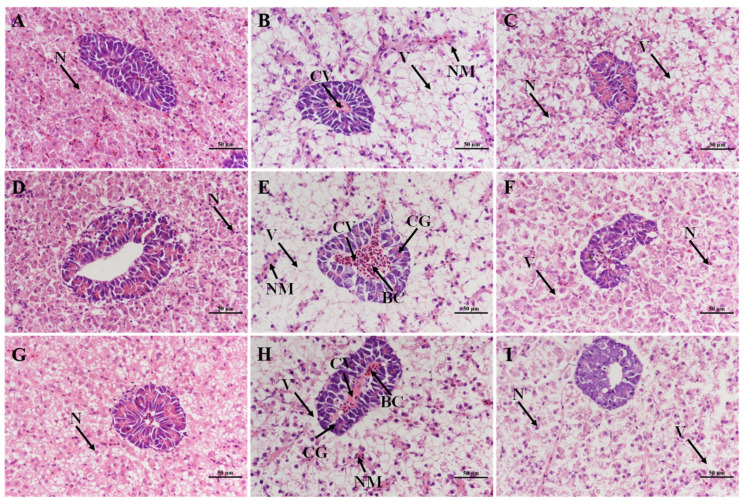
Effects of the compound feed additive on histological changes in the liver of Nile tilapia under saline–alkaline stress: (**A**–**C**) TW diet group; (**D**–**F**) HX diet group; (**G**–**I**) DBN diet group. From left to right: FW, SAW, and SAW+CFA treatments. Respectively, under saline-alkali stress, liver tissue exhibited central vein (CV) dilation, erythrocyte congestion, increased hepatocyte lipid vacuoles (V), and disorganized tissue structure (**B**,**E**,**H**). Supplementation with compound feed additives under saline-alkali water conditions significantly alleviated the above histological damage, and the arrangement of hepatocytes became regular. N: nucleus; CV: central vein; V: lipid vacuole; NM: nuclear membrane; CG: hepatic glycogen; BC: bile canaliculus. Scale bar = 50 μm.

To evaluate the effects of saline–alkaline stress and dietary additive intervention on hepatic tissue structure, liver samples from each treatment group were subjected to hematoxylin–eosin (H&E) staining (Figure 4). In the FW group, the hepatic architecture was intact, with normal morphology of the central vein. Hepatocytes were closely and orderly arranged, with clear cell boundaries and regularly shaped nuclei. The hepatic cords were well defined, and no obvious pathological alterations were observed (Figure 4A,D,G). Compared with the FW group, the SAW group exhibited marked histopathological abnormalities. These were mainly characterized by dilation of the central vein, local aggregation of erythrocytes and congestion, and varying degrees of lipid vacuolation within hepatocytes, suggesting hepatic steatosis and congestion (Figure 4B,E,H). In contrast, the SAW+CFA treatment group show significant improvement in hepatic histological structure compared with the SAW group. Central vein dilation and congestion were alleviated, the number of lipid vacuoles in hepatocytes was markedly reduced, and hepatocyte arrangement became more regular. Overall tissue integrity was partially restored (Figure 4C,F,I).

### 3.5. Hepatic Antioxidant Capacity and Apoptosis Level

**Figure 5:**
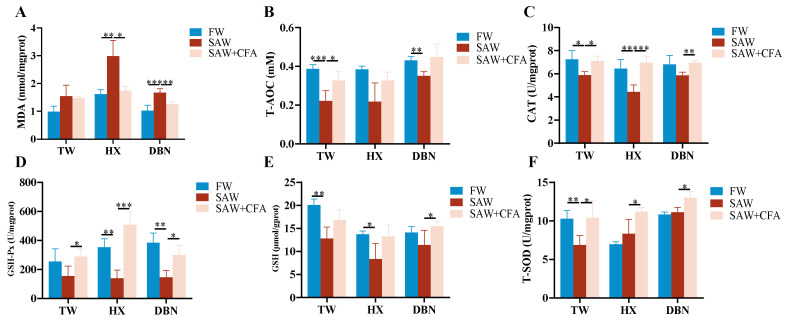
Effects of the compound feed additive on hepatic antioxidant capacity of Nile tilapia under saline–alkaline stress: (**A**) malondialdehyde (MDA) content; (**B**) total antioxidant capacity (T-AOC); (**C**) catalase (CAT) activity; (**D**) glutathione peroxidase (GSH-Px) activity; (**E**) GSH level; (**F**) total superoxide dismutase (T-SOD) activity. The definitions of FW, SAW, and SAW+CFA groups are the same as described previously. Data are presented as mean ± SD (*n* = 3). Statistical differences between treatment groups under the same basal diet were analyzed using independent-samples *t*-tests. Significance levels are indicated as * *p* < 0.05, ** *p* < 0.01, *** *p* < 0.001.

**Figure 6:**
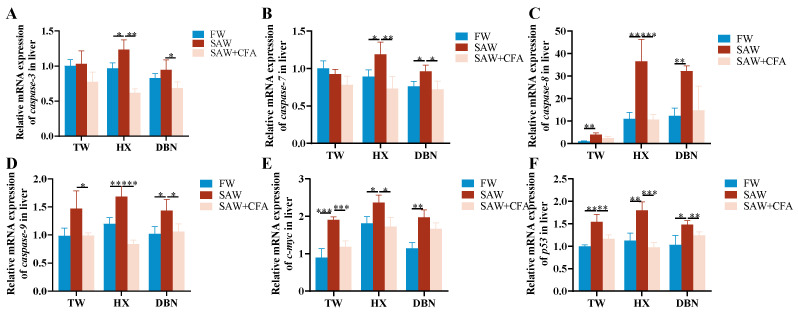
Effects of the compound feed additive on relative expression levels of apoptosis-related genes in the liver of Nile tilapia under saline–alkaline stress: (**A**–**D**) relative mRNA expression levels of caspase family genes: (**A**) *caspase-3*, (**B**) *caspase-7*, (**C**) *caspase-8*, and (**D**) *caspase-9*; (**E**,**F**) relative mRNA expression levels of (**E**) *c-myc* and (**F**) *p53*. Gene expression levels were determined by qRT-PCR and normalized to *β-actin* and *EF-1α* as reference genes. FW represents the freshwater group, SAW represents the saline–alkaline water group, and SAW+CFA represents the saline–alkaline water group supplemented with the compound feed additive. Data are presented as mean ± SD (*n* = 3). Differences between treatment groups under the same basal diet were analyzed using independent-samples *t*-tests. Significance levels are indicated as * *p* < 0.05, ** *p* < 0.01, *** *p* < 0.001.

To evaluate the effects of saline–alkaline stress on hepatic oxidative damage and the antioxidant defense system in Nile tilapia, as well as the mitigating role of the compound feed additive, multiple antioxidant parameters were measured in liver tissues. Compared with the FW group, hepatic malondialdehyde (MDA) content was significantly increased in the SAW group, whereas total antioxidant capacity (T-AOC) was markedly decreased (Figure 5A,B), indicating pronounced oxidative damage induced by saline–alkaline stress. Meanwhile, the activities of catalase (CAT), glutathione peroxidase (GSH-Px), and GSH level was significantly reduced in the SAW group, and total superoxide dismutase (T-SOD) activity also showed a declining trend (Figure 5C–F), suggesting suppression of the hepatic antioxidant defense system. In the SAW+CFA treatment group, hepatic MDA levels were significantly lower than those in the SAW group, while T-AOC levels were markedly restored (Figure 5A,B). Concurrently, CAT, GSH-Px, and GSH content was significantly elevated, and T-SOD activity was recovered (Figure 5C–F). These results indicate that saline–alkaline stress markedly induces oxidative stress and impairs antioxidant capacity in the liver of Nile tilapia, whereas supplementation with the compound feed additive effectively alleviates oxidative damage and enhances hepatic antioxidant defense. To further assess the effects of saline–alkaline stress and dietary supplementation on hepatic apoptosis, the transcriptional levels of multiple apoptosis-related genes were analyzed by qRT-PCR. Compared with the FW group, the mRNA expression levels of *caspase-3*, *caspase-7*, *caspase-8*, and *caspase-9* in the liver were significantly upregulated in the SAW group, showing consistent trends under different basal diet conditions (Figure 6A–D). Following supplementation with the compound feed additive under saline–alkaline conditions (SAW+CFA), the expression levels of these caspase-related genes were markedly reduced compared with the SAW group, with some indices returning to levels close to those of the FW group (Figure 6A–D). In addition, saline–alkaline stress significantly upregulated the hepatic transcription of *c-myc* and *p53* (Figure 6E,F). Compared with the SAW group, the expression of both genes was downregulated to varying extents in the SAW+CFA group (Figure 6E,F), indicating that the compound feed additive effectively mitigates the abnormal activation of apoptosis-related genes induced by saline–alkaline stress in the liver.

### 3.6. Histological Observation of the Gill

**Figure 7:**
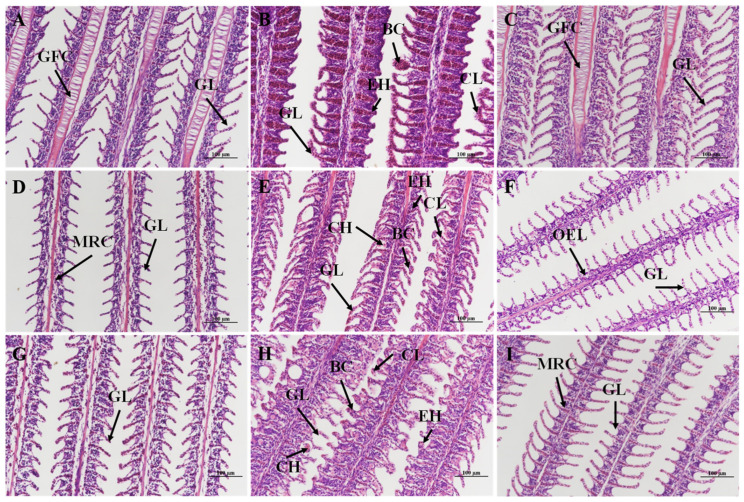
Effects of the compound feed additive on gill histomorphology of Nile tilapia under saline–alkaline stress**:** (**A**–**C**) TW basal diet group ((**A**) FW; (**B**) SAW; (**C**) SAW+CFA); (**D**–**F**) HX basal diet group ((**D**) FW; (**E**) SAW; (**F**) SAW+CFA); (**G**–**I**) DBN basal diet group ((**G**) FW; (**H**) SAW; (**I**) SAW+CFA). Under freshwater conditions (FW), the gill structure was intact, with orderly arranged filaments and slender, regularly shaped secondary lamellae. Under saline–alkaline stress (SAW), typical pathological alterations were observed, including curling of secondary lamellae, shortening of filaments, erythrocyte aggregation, chloride cell hypertrophy, and epithelial hyperplasia. Following supplementation with the compound feed additive under saline–alkaline conditions (SAW+CFA), these pathological lesions were markedly alleviated. GL gill lamella; MRC: mitochondria-rich cell; OEL: outer epithelial layer; BC: blood cell; CH: chlorine cell hypertrophy; GFC: gill filament cartilage; EH: epithelial hyperplasia; CL: curling of secondary lamella. Scale bar = 100 μm.

Under freshwater conditions, fish from different basal diet groups exhibited intact gill architecture, with orderly arranged gill filaments and slender, regularly shaped secondary lamellae. The epithelial structure was clearly defined, and no obvious pathological alterations were observed (Figure 7A,D,G). Under saline–alkaline stress (SAW), marked structural damage was observed in the gill tissues. The primary pathological features included curling of the secondary lamellae, shortening of filaments, erythrocyte aggregation, hypertrophy of chloride cells, and epithelial hyperplasia (Figure 7B,E,H), indicating that saline–alkaline stress severely disrupted normal gill morphology. In contrast, supplementation with the compound feed additive under saline–alkaline conditions (SAW+CFA) markedly alleviated these histopathological alterations. The secondary lamellae appeared more regular with reduced curling, the epithelial structure was relatively preserved, and both erythrocyte aggregation and chloride cell hypertrophy were attenuated (Figure 7C,F,I). Collectively, these results demonstrate that the compound feed additive partially mitigates structural damage in gill tissue induced by saline alkaline stress and contributes to the maintenance of gill morphological integrity.

### 3.7. Osmoregulation, Ion Transport, and Ammonia Metabolism

**Figure 8:**
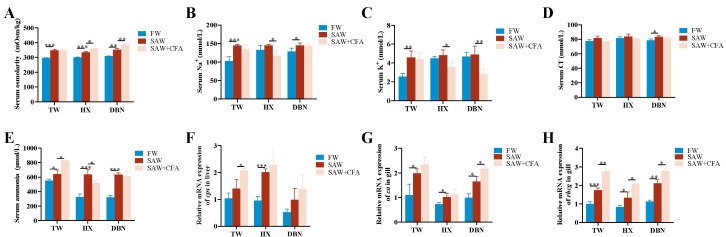
Effects of compound feed additives on osmoregulation and ammonia metabolism in Nile tilapia under saline–alkaline stress: (**A**) serum osmolality; (**B**–**E**) serum contents of Na^+^, K^+^, Cl^−^ and blood ammonia; (**F**–**H**) relative mRNA expression levels of ammonia metabolism and transport related genes. (**F**) *cps*, (**G**) *ca*, (**H**) *rhcg*. FW: Freshwater group; SAW: Saline alkali water group; SAW+CFA: Saline alkali water + compound feed additives group. Data are presented as mean ± SD (*n* = 3). Different symbols indicate significant differences between groups (* *p* < 0.05, ** *p* < 0.01, *** *p* < 0.001).

**Figure 9:**
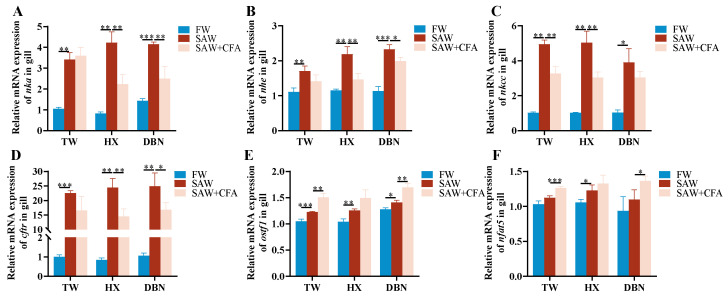
Effects of compound feed additives on the expression of ion transport-related genes in Nile tilapia under saline–alkaline stress. Relative mRNA expression levels of ion transport-related genes in gill tissue: (**A**) *nka*, (**B**) *nhe*, (**C**) *nkcc*, (**D**) *cftr*, (**E**) *ostf1*, (**F**) *nfat5.* FW: Freshwater group; SAW: Saline-alkali water group; SAW+CFA: Saline-alkali water + compound feed additives groups. Data are presented as mean ± SD (*n* = 3). * *p* < 0.05, ** *p* < 0.01, *** *p* < 0.001.

To systematically elucidate the physiological regulatory mechanisms underlying internal homeostasis in fish under saline–alkaline stress, serum osmolality and inorganic ion levels, the expression of ion transport–related genes in gill tissues, and parameters associated with ammonia metabolism and transport were comprehensively analyzed. Compared with the FW group, fish in the SAW group exhibited a significant increase in serum osmolality, accompanied by markedly elevated serum Na^+^, K^+^, and Cl^−^ concentrations, indicating substantial accumulation of inorganic ions under saline–alkaline stress. This ionic imbalance increased the osmotic load and may have contributed to ion toxicity (Figure 8A–D). In addition, saline–alkaline stress significantly affected nitrogen metabolism. Serum ammonia levels were markedly elevated in the SAW group, along with significant alterations in the expression of genes involved in ammonia metabolism and transport, suggesting activation of ammonia metabolic and excretory pathways under stress conditions (Figure 8E–H). Furthermore, saline–alkaline stress significantly upregulated the expression of multiple genes associated with ion transport and osmoregulation in gill tissues, indicating that fish enhanced branchial ion transport capacity to counteract osmotic imbalance induced by the high saline–alkaline environment (Figure 9A–F).

Notably, compared with the SAW group, supplementation with the compound feed additive resulted in an overall reduction in serum inorganic ion levels and downregulation of ion transport–related gene expression (Figure 8B–D and Figure 9A–F), suggesting alleviation of ion toxicity. Meanwhile, the compound feed additive further increased systemic osmolality and enhanced the expression of genes involved in ammonia metabolism and transport (Figure 8A,E–H). Collectively, these findings suggest that the compound feed additive may improve the adaptive capacity of fish under saline–alkaline stress through the modulation of multiple physiological processes, including ion balance and nitrogen metabolism.

### 3.8. Intestinal Morphology, Digestive Capacity, and Inflammatory Response

**Figure 10:**
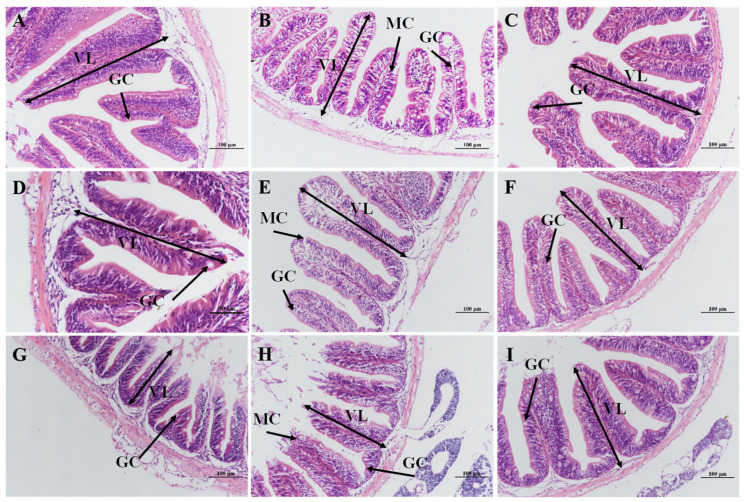
Effects of the compound feed additive on intestinal health of Nile tilapia under saline–alkaline stress. Histological changes in intestinal tissues (H&E staining): (**A**–**C**) TW basal diet group ((**A**) FW; (**B**) SAW; (**C**) SAW+CFA); (**D**–**F**) HX basal diet group ((**D**) FW; (**E**) SAW; (**F**) SAW+CFA); (**G**–**I**) DBN basal diet group ((**G**) FW; (**H**) SAW; (**I**) SAW+CFA). Under saline–alkaline stress (SAW), typical pathological alterations were observed in the intestine, including villus atrophy, disorganized arrangement, goblet cell hyperplasia, and degeneration of mucosal epithelial cells. Following supplementation with the compound feed additive under saline–alkaline conditions (SAW+CFA), these intestinal lesions were markedly alleviated, and villus architecture appeared relatively restored. GC: goblet cells, VL: length of intestinal villus, MC: intestinal mucosal cell degeneration. Scale bar = 100 μm.

Saline–alkaline stress markedly impaired intestinal histological structure in fish. In the SAW group, obvious pathological alterations were observed, including pronounced villus atrophy, disorganized arrangement, a significant increase in goblet cell number, and varying degrees of degeneration of intestinal mucosal epithelial cells, indicating that saline–alkaline stress adversely affected the integrity of the intestinal barrier (Figure 10B,E,H). In contrast, supplementation with the compound feed additive under saline–alkaline conditions significantly alleviated these histopathological changes, as evidenced by increased villus length, improved tissue organization, and marked attenuation of goblet cell hyperplasia and epithelial cell degeneration (Figure 10C,F,I). Quantitative analysis of villus length further supported the histological observations (Table 4).

**Figure 11:**
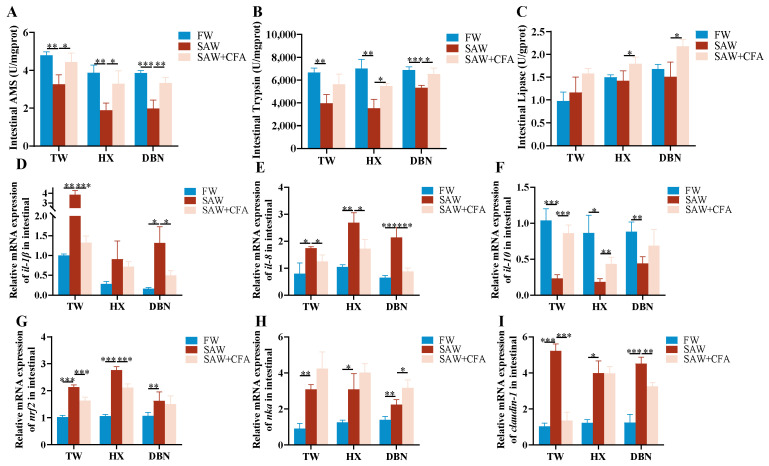
Effects of compound feed additives on intestinal digestive function and inflammatory response in Nile tilapia under saline-alkali stress. (**A**–**C**) Changes in intestinal digestive enzyme activities, including (**A**) α-amylase, (**B**) trypsin, and (**C**) lipase activities. (**D**–**I**) Relative mRNA expression levels of genes related to intestinal inflammatory response and barrier function: (**D**) *il-1β*, (**E**) *il-8*, (**F**) *il-10*, (**G**) *nrf2*, (**H**) *nka*, and (**I**) *claudin-1*. FW represents the freshwater group, SAW represents the saline-alkali water group, and SAW+CFA represents the saline-alkali water group supplemented with compound feed additives. Data are presented as the mean ± SD (*n* = 3). Differences among different treatment groups under the same basal diet were analyzed by independent-sample *t*-test. Significance levels are indicated as * *p* < 0.05, ** *p* < 0.01, *** *p* < 0.001.

At the functional level, saline–alkaline stress significantly reduced intestinal digestive enzyme activities (Figure 11A–C). Compared with the FW group, the activities of amylase, trypsin, and lipase were significantly decreased in the SAW group, indicating suppression of intestinal digestive capacity under stress conditions. Following supplementation with the compound feed additive, the activities of these enzymes were significantly increased, with some indices restored to or approaching FW levels, suggesting improved digestive function under saline–alkaline stress. At the molecular level, saline–alkaline stress significantly altered the expression of genes associated with intestinal inflammation and barrier function (Figure 11D–I). In the SAW group, the mRNA expression levels of pro-inflammatory genes *il-1β* and *il-8*, as well as *nrf2*, were significantly upregulated, whereas the anti-inflammatory cytokine *il-10* was significantly downregulated. In addition, the expression levels of intestinal barrier–related genes *claudin-1* and *nka* were significantly increased. Compared with the SAW group, supplementation with the compound feed additive markedly suppressed the abnormal upregulation of pro-inflammatory genes and enhanced the expression of anti-inflammatory and barrier-related genes. These findings suggest that the compound feed additive alleviates saline–alkaline stress–induced intestinal injury by modulating inflammatory responses and maintaining intestinal barrier function, thereby improving intestinal health in fish.

## 4. Discussion

Saline–alkaline environments markedly inhibit the normal ammonia excretion capacity of aquatic organisms. This inhibition further disrupts amino acid metabolic pathways and ultimately exerts negative effects on growth performance in aquatic animals [38]. Previous studies have shown that at a carbonate alkalinity of 15 mmol/L, the growth performance and feed utilization of Songpu mirror carp (*Cyprinus carpio Songpu*) were significantly restricted, as evidenced by a marked decrease in weight gain rate (WGR) and a significant increase in feed conversion ratio (FCR). These findings are consistent with the present study, in which saline–alkaline stress significantly reduced growth and survival rates in tilapia, increased FCR, and decreased whole-body crude protein content. These effects may be attributed to the substantial energy expenditure required to maintain acid–base balance and osmotic homeostasis under saline–alkaline stress, thereby reducing the energy available for growth and development [39]. Under such stress conditions, carbohydrates serve as the primary energy source for aquatic animals and play a crucial role in direct energy supply. During environmental challenges, carbohydrate metabolism not only sustains basic physiological functions but also provides essential energy support for stress adaptation [40]. Glucose, as a preferred energy substrate, can largely meet the energetic demands associated with osmoregulation and acid–base balance in fish [41]. Accordingly, in the present study, saline–alkaline stress significantly enhanced glycolysis, gluconeogenesis, and the pentose phosphate pathway, accompanied by elevated serum glucose levels in tilapia, presumably to meet increased energy demands under stress. Meanwhile, endogenous proteins may also be converted into glucose via gluconeogenesis to provide additional energy support. Notably, dietary supplementation with the compound additive significantly alleviated the adverse effects of saline–alkaline stress on growth performance. Further analysis revealed that supplementation with the compound additive enhanced gluconeogenesis and the pentose phosphate pathway while suppressing glycolysis, promoted glycogen synthesis, and increased crude protein content. These findings are consistent with previous studies demonstrating that several bioactive components included in the CFA, particularly glutamate and myo-inositol, contribute to carbohydrate utilization, protein deposition, and physiological adaptation to environmental stress in fish. For example, glutamate plays an important role in growth, feed utilization, and protein synthesis in common carp (*Cyprinus carpio*) [42]. In addition, dietary supplementation with myo-inositol (MI) under salinity stress has been shown to promote carbohydrate utilization in Nile tilapia, thereby enhancing osmoregulatory capacity and growth performance.

Carbonate alkalinity stress and ammonia toxicity can both induce excessive production of reactive oxygen species (ROS), thereby disrupting antioxidant defense systems and inhibiting normal growth and development in fish [43,44]. Malondialdehyde (MDA), a typical end product of lipid peroxidation, is widely recognized as an important indicator of oxidative damage, and saline–alkaline stress has been shown to significantly increase MDA levels in fish [45]. The antioxidant defense system in fish mainly consists of enzymatic components, including catalase (CAT), total superoxide dismutase (T-SOD), Cu/Zn-superoxide dismutase (Cu/Zn-SOD), and glutathione peroxidase (GSH-Px), as well as the non-enzymatic antioxidant glutathione (GSH). Together, these systems scavenge ROS and maintain intracellular redox homeostasis [46]. However, when ROS production exceeds the antioxidant scavenging capacity, oxidative stress occurs, resulting in damage to proteins, lipids, and DNA, and subsequently triggering apoptotic pathways. In the present study, saline–alkaline stress significantly increased ROS and MDA levels, suppressed antioxidant enzyme activities, and upregulated apoptosis-related gene expression. In contrast, supplementation with the compound additive markedly reduced ROS and MDA levels, enhanced antioxidant enzyme activities, and inhibited apoptosis. These protective effects may be associated with the combined presence of multiple bioactive components in the additive. Previous studies have shown that glutamate can alleviate copper-induced oxidative damage by modulating antioxidant defenses; β-glucan exhibits free radical scavenging activity; curcumin significantly improves growth performance, antioxidant status, and immune function in teleost fish; and zinc, as a cofactor of Cu/Zn-SOD—particularly in the form of zinc methionine (Zn-Met)—is considered an effective zinc source for enhancing growth and immunity in fish [47,48].

The toxic effects of saline–alkaline conditions are generally attributed to the combined influences of osmotic pressure, high pH, and carbonate alkalinity [49,50]. As the primary organ directly exposed to the aquatic environment, the gill is widely regarded as a sensitive indicator organ in aquatic toxicology studies [51]. Under carbonate–alkaline conditions, gill tissue is particularly susceptible to structural damage. In addition, saline–alkaline stress is known to disrupt ionic homeostasis, including Na^+^, K^+^, and Cl^−^ balance, thereby impairing osmotic regulation. It has been reported that Na^+^/K^+^-ATPase (NKA) is a key energy-dependent ion pump involved in osmoregulation in fish, while Rhesus glycoprotein C (*rhcg*) mediates ammonia transport across membranes. Carbamoyl phosphate synthetase (CPS) converts toxic ammonia into relatively non-toxic urea to facilitate ammonia excretion. Carbonic anhydrase (CA) also plays a crucial role in maintaining acid–base balance in the gills by catalyzing the conversion of CO_2_ into HCO_3_^−^ and H^+^, thereby providing necessary conditions for ammonia excretion [52,53]. Disturbances in ion homeostasis and acid–base balance induced by saline–alkaline stress, particularly the deficiency of H^+^, can significantly inhibit ammonia excretion and lead to plasma ammonia accumulation. Therefore, saline–alkaline waters generally exert negative effects on osmoregulation and ammonia metabolism in fish [54]. Fish possess two major ammonia detoxification pathways. The glutamine synthesis pathway is considered one of the key mechanisms enabling fish to adapt to alkaline environments. In this pathway, glutamine synthetase (GS) catalyzes the combination of ammonia and glutamate to form non-toxic glutamine, thereby effectively reducing ammonia toxicity. The other pathway is the urea synthesis pathway, which converts ammonia into urea, a relatively non-toxic and readily excretable compound, thus helping maintain ammonia homeostasis and alleviating toxicity under alkaline conditions [55]. In the present study, saline–alkaline stress significantly damaged gill structure in tilapia and induced upregulation of ion transport-related genes, resulting in increased accumulation of inorganic ions and subsequent ionic toxicity. Meanwhile, serum ammonia concentrations were significantly higher in the saline–alkaline group than in the freshwater group, and the expression levels of *cps*, *ca*, and *rhcg* were markedly upregulated, indicating activation of ammonia metabolism under stress. Supplementation with the compound additive markedly alleviated gill damage, reduced ion transporter gene expression and inorganic ion accumulation, thereby mitigating ionic toxicity. In addition, the additive further enhanced the expression of ammonia metabolism- and transport-related genes, improved ammonia metabolic capacity, and alleviated ammonia toxicity. These effects may reflect the complementary functions of multiple bioactive components within the CFA, particularly those involved in osmotic regulation and membrane stabilization. These effects may be associated with glutamate and MI in the additive acting as organic osmolytes to enhance osmoregulatory capacity. Previous studies have shown that dietary MI supplementation under salinity stress significantly enhances gill ion transport capacity and reduces serum ion concentrations in tilapia. Furthermore, cholesterol supplementation can directly enhance NKA activity and improve osmoregulatory capacity in *Litopenaeus vannamei* [28].

When seawater carbonate alkalinity exceeds 10 mmol/L, digestive enzyme activities in Japanese seabass (*Lateolabrax japonicus*) significantly decrease [56]. Similarly, at a carbonate alkalinity of 15 mmol/L, amylase and lipase activities in the intestine of *Songpu mirror* carp were significantly reduced [38]. Consistent with these findings, the present study demonstrated that saline–alkaline stress significantly decreased intestinal digestive enzyme activities in tilapia. This reduction may be attributed to elevated environmental pH exceeding the optimal range for digestive enzyme activity, thereby inhibiting enzymatic function and reducing digestive and absorptive capacity as well as feed intake [57]. Supplementation with the compound additive significantly enhanced digestive enzyme activities and alleviated intestinal damage. Previous studies have also shown that cholesterol supplementation in soybean meal-based diets significantly improves digestive enzyme activities and promotes nutrient absorption in tilapia [34]. In addition, intestinal immune function in fish primarily depends on cytokine regulation and the integrity of the physical barrier. Cytokines include pro-inflammatory factors (*il-1β* and *il-8*) and anti-inflammatory factors (*il-10*), and changes in their expression directly influence the occurrence and progression of inflammation [58]. The present results showed that saline–alkaline stress significantly upregulated intestinal pro-inflammatory cytokines (*il-1β* and *il-8*) while suppressing anti-inflammatory cytokine (*il-10*) expression, indicating the induction of intestinal inflammation. Supplementation with the compound additive effectively inhibited the abnormal elevation of pro-inflammatory cytokines and upregulated anti-inflammatory cytokine expression, thereby alleviating intestinal injury and improving intestinal health. These beneficial effects may result from the combined antioxidant, anti-inflammatory, and intestinal protective properties of the bioactive components included in the CFA. Previous studies have shown that glutamate alleviates LPS-induced oxidative damage by activating the Nrf2 signaling pathway and improves growth, digestion, and intestinal barrier function in fish [59]. Dietary curcumin supplementation has also been reported to mitigate inflammation by downregulating pro-inflammatory genes and upregulating anti-inflammatory genes [48].

The present results showed that CFA supplementation was associated with improvements in multiple physiological processes under saline–alkaline stress. Enhanced carbohydrate utilization and glycogen storage may provide sufficient energy to support ion transport and osmoregulatory functions. Improved antioxidant capacity likely reduced oxidative damage in metabolically active tissues, thereby limiting apoptosis and preserving cellular function. Meanwhile, maintenance of gill integrity facilitated ion balance and ammonia excretion, whereas improved intestinal health promoted nutrient digestion and absorption. Overall, CFA supplementation was associated with improvements in energy metabolism, oxidative homeostasis, osmoregulation, ammonia metabolism, and intestinal function under saline–alkaline stress. These changes likely reflect the combined contributions of the bioactive components within the formulation. Importantly, similar response patterns were observed across the three dietary systems, suggesting that the beneficial effects of CFA were largely independent of the basal diet formulation and may therefore have broader applicability in saline–alkaline aquaculture. Although the three commercial basal diets differed modestly in crude protein and crude lipid contents, CFA consistently improved physiological performance across all dietary backgrounds. This finding suggests that its protective effects are relatively robust to moderate variations in basal nutrient composition. Rather than relying on a specific nutritional profile, the additive appears to modulate common stress-responsive pathways, including energy metabolism, antioxidant defense, osmoregulation, and intestinal function, thereby supporting its potential versatility for application in commercially available tilapia feeds [60,61].

Although the CFA was formulated to address multiple physiological disturbances associated with saline–alkaline stress, potential functional overlap among its components should be acknowledged. For example, certain ingredients, such as glutamate and curcumin, may partially converge on antioxidant and anti-inflammatory pathways. Therefore, it remains unclear whether all six components are required to achieve the observed physiological benefits or whether a reduced formulation could provide comparable efficacy. From a practical perspective, the inclusion of multiple bioactive ingredients may also increase formulation costs and implementation complexity. Although the present study did not include a formal cost–benefit analysis, CFA supplementation consistently improved feed utilization and survival under saline–alkaline conditions. Because feed efficiency and survival are major determinants of production profitability in aquaculture, these improvements may partially offset the additional costs associated with the inclusion of multiple bioactive ingredients [62]. Nevertheless, further economic evaluation under commercial farming conditions is required to determine whether the observed biological benefits ultimately translate into net economic gains. Future studies should therefore evaluate both biological efficacy and economic performance under commercial farming conditions to identify the most cost-effective formulation for large-scale application.

Several limitations of the present study should also be considered. First, only the complete CFA formulation was evaluated, and individual ingredients or partial combinations were not tested. Consequently, the relative contribution of each component and potential interactions among components could not be determined. Second, histological assessments were primarily based on qualitative observations, and no quantitative histomorphometric or histopathological scoring analyses were conducted. Third, the feeding trial lasted for only 42 days and therefore evaluated only the short-term effects of CFA supplementation under saline–alkaline conditions. Potential long-term consequences, including tissue accumulation, lipid deposition, mineral retention, or chronic metabolic burdens associated with prolonged dietary supplementation, were not assessed [63,64]. Future studies incorporating component-deletion or fractional factorial experimental designs, longer-term feeding trials, and quantitative tissue analyses will be necessary to clarify the functional roles of individual ingredients, evaluate long-term safety and efficacy, and further strengthen mechanistic interpretation.

## 5. Conclusions

In summary, saline–alkaline stress significantly impairs growth performance and survival in aquatic animals, weakens antioxidant capacity, and disrupts osmoregulation, ammonia metabolism, and intestinal health. Incorporating the compound feed additive into tilapia diets under saline–alkaline aquaculture conditions effectively improve growth, survival, feed utilization, antioxidant and anti-inflammatory capacities, enhances intestinal digestive and absorptive functions, and strengthens osmoregulatory and ammonia metabolic capacity. Therefore, this compound feed additive shows promising application prospects and practical value in saline–alkaline aquaculture of tilapia.

## Figures and Tables

**Table 1:** Composition and inclusion levels of the compound feed additive (CFA).

Ingredient	Inclusion Level (g/kg Diet)
Glutamate	7.79 [33]
Cholesterol	8.3 [34]
β-glucan	0.5 [27]
Inositol	0.401 [35]
Zinc methionine	0.3 [36]
Curcumin	0.2 [37]

**Table 2:** Nutritional composition of commercial basal diets.

Nutrient	TW	HX	DBN
Crude protein (%)	31	30	28
Crude lipid (%)	3.5	3.0	4.5

Note: Values are expressed as percentage of dry matter basis.

**Table 3:** Primer pair sequences of genes used for real-time PCR (qPCR).

Gene	Sequence ID	Primer	Primer Sequences (5′–3′)	Product Size (bp)
*pk*	XM_005472622.4	F	CAGCATAATCTGCACCATCGGT	99
		R	ATGAGAGAAGTTAAGACGGGCGA	
*pfk1*	XM_003441476.5	F	CTGACATGACCATCGGCACT	139
		R	CACCAAAGCCAGGTAACCAC	
*hk*	XM_039611531.1	F	TTCCTCTGGGCTTCACCTTCT	109
		R	ATCTTCCCCTTCGCAGTCTGT	
*g6pase*	XM_003448671.5	F	GGATGCTAATGGGCCTGGTC	110
		R	CAGCTACCAGTGTGCCTGTAA	
*pc*	XM_003452415.5	F	ATGTCACACCCGATGCTTCC	108
		R	ATATCGTCTGAACGCCTGCC	
*gys*	XM_013276796.3	F	CCTCACTCTGCGCTGTTATTC	99
		R	CAGCGGCATGCCTTCAGTTT	
*g6pdh*	DQ066877.1	F	TCCAGAACCTCATGGTGCTT	96
		R	GGCTCCTTGAAGGTAAGGACG	
*nka*	XM_005452357.3	F	TTCCCCACTGAGAACTTGTGC	160
		R	ACACCTTTAGCGATGGCCTTG	
*nhe*	XM_005455318.4	F	AGAGAGCTGCCTGTTGATCGT	116
		R	GGGGGCAGCAGGATGTAAAAA	
*nkcc*	XM_003439466.5	F	GCAAGATGAGTCGCTGTCCT	150
		R	ATCTTTCTGGACTGCTGGGC	
*cftr*	XM_003449170.5	F	AACAGCCAGGATCGTGAACAG	158
		R	GGGAGAGCAGCGATGAAGATG	
*ostf1*	XM_003451065.5	F	CTTTATCCGCCAGCCTCACA	243
		R	TGTCGATAGACTCTGCCTGC	
*nfat5*	XM_005470887.4	F	TCCCTCAGAGGAGTGATGGG	192
		R	CTGCCCGCATCATTTGCTAC	
*caspase3*	NM_001282894.1	F	GGAGTGGACGATACAGACGCAAA	108
		R	TGAAGCTGTGTGACTGGGGCTT	
*caspase7*	XM_013269445.3	F	GTGAACCCTCTGAACTTGGAG	87
		R	AGAAATCTCCCTTTGCGGTCA	
*caspase8*	XM_005475926.4	F	AACTGGAATCTTCTGAGGTGGCA	101
		R	TTGAGAAGAGATCTTTGGCCTGC	
*caspase9*	XM_025901776.1	F	GAGAGTTTGGCAGAGCTCCTA	104
		R	GGGAATTGGAAGAGGCTGGAT	
*p53*	XM_025905405.1	F	GCATGTGGCTGATGTTGTTC	190
		R	GCGCCGCAGATCTTATTGTG	
*c-myc*	XM_019345277.2	R	AACCCTGCTTTAGACGCTCC	99
		R	GCAGGATGGTGGTCATCTCT	
*rhcg*	XM_003440579.4	F	CCAGGATGTCCATGTGATGATATTT	187
		R	TTTCAATTCCAATTTTGATCTTCCC	
*gclc*	XM_003441123.5	F	GCAGCATTGATGAGGCAAGG	96
		R	TGGCCTCTGTAGAAGGGTGA	
*gs*	NM_001279668.1	F	GTCGCCCTTCAGCCAACT	87
		R	GCGGGTTCATCTCCTTCC	
*cps*	XM_003445297.5	F	GGTCAGGCTGGTGAGTTT	101
		R	ACAGAGGCTATGTTTGGATT	
*ca*	XM_005448311.3	F	AGCATACAGTGGATGGAAAGCG	155
		R	GACCAGTTGAGTTGCCTGACATT	
*il-1β*	XM_019365842.2	F	GAGCACAGAATTCCAGGATGAAAG	100
		R	TGAACTGAGGTGGTCCA GCTGT	
*il-8*	NM_001279704.1	F	CTGTGAAGGCATGGGTGTGGAG	110
		R	TCGCAGTGGGAGTTGGGAAGAA	
*il-10*	KP645180.1	F	CAGCAGCAGGAGCATCAGCATT	107
		R	CACAGGAGGACGGTCTGAGAAGT	
*nrf2*	XM_003447296.5	F	CCTGGAGGTCTTTGGCATGT	81
		R	ATCCGTTGACTGCTGAAGGG	
*claudin1*	XM_019367708.2	F	GAGGAGTCAGTCGGAGTCT	114
		R	CAGCACCGTCTTGAACTTG	
*EF-1α*	NM_001279647.1	F	ATCAAGAAGATCGGCTACAACCCT	109
		R	ATCCCTTGAACCAGCTCATCTTGT	
*β-actin*	AY116536.1	F	GGATTCACTCTGAGCGCCG	105
		R	CCGTCTCCTTACCTTTGGGTG	

Note: pk = pyruvate kinase; pfk1 = phosphofructokinase-1; hk = hexokinase; g6pase = glucose-6-phosphatase; pc = pyruvate carboxylase; gys = glycogen synthase; g6pdh = glucose-6-phosphate dehydrogenase; nka = Na^+^/K^+^-ATPase; nhe = Na^+^/H^+^ exchanger; nkcc = Na^+^/K^+^/2Cl^−^ cotransporter; cftr = cystic fibrosis transmembrane conductance regulator; ostf1 = osteoclast stimulating factor 1; nfat5 = nuclear factor of activated T cells 5; caspase3 = caspase 3; caspase7 = caspase 7; caspase8 = caspase 8; caspase9 = caspase 9; p53 = tumor protein p53; c-myc = MYC proto-oncogene; rhcg = Rh type C glycoprotein; gclc = glutamate–cysteine ligase catalytic subunit; GS = glutamine synthetase; cps = carbamoyl phosphate synthetase; ca = carbonic anhydrase; il-1β = interleukin-1 beta; il-8 = interleukin-8; il-10 = interleukin-10; nrf2 = nuclear factor erythroid 2–related factor 2; claudin1 = claudin-1; EF-1α = elongation factor 1 alpha; β-actin = beta-actin.

**Table 4:** Quantitative analysis of intestinal villus length.

	TW (μm)	HX (μm)	DBN (μm)
FW	446.4 ± 10.2 ^a^	455.0 ± 11.4 ^a^	236.8 ± 18.5 ^b^
SAW	321.8 ± 14.9 ^b^	349.6 ± 37.1 ^b^	264.9 ± 7.6 ^b^
SAW+CFA	371.6 ± 31.4 ^ab^	352.2 ± 11.8 ^b^	334.0 ± 8.3 ^a^

Note: Statistical differences were analyzed using Student’s *t*-test. Different superscript letters within the same column indicate significant differences (*p* < 0.05). Data are presented as mean ± SD.

## Data Availability

The original contributions presented in this study are included in the article. Further inquiries can be directed to the corresponding author.
